# Draft Genome Sequences of 25 *Listeria monocytogenes* Isolates Associated with Human Clinical Listeriosis in Ireland

**DOI:** 10.1128/genomeA.00184-17

**Published:** 2017-05-11

**Authors:** Amy O’Callaghan, Amber Hilliard, Ciara A. Morgan, Eamonn P. Culligan, Dara Leong, Niall DeLappe, Colin Hill, Kieran Jordan, Martin Cormican, Cormac G. M. Gahan

**Affiliations:** a APC Microbiome Institute, University College Cork, Cork, Ireland; b School of Microbiology, University College Cork, Cork, Ireland; c Teagasc Food Research Centre, Moorepark, Fermoy, Cork, Ireland; d National Salmonella, Shigella and Listeria Reference Laboratory, Galway University Hospitals, Galway, Ireland; e School of Medicine, National University of Ireland, Galway, Ireland; f School of Pharmacy, University College Cork, Cork, Ireland

## Abstract

*Listeria monocytogenes* is a Gram-positive opportunistic pathogen that is the causative agent of listeriosis. Here, we report the draft genome sequences of 25 *L. monocytogenes* strains isolated from patients with clinical listeriosis in the Republic of Ireland between 2013 and 2015.

## GENOME ANNOUNCEMENT

*Listeria monocytogenes* is a Gram-positive, intracellular foodborne pathogen that causes listeriosis. Contaminated foods, in particular, ready-to-eat foods, are the primary vehicle of transmission to humans. Infections can result in mild gastroenteritis in otherwise healthy individuals. However, more common presentations of the disease are invasive infections such as bloodstream infection, meningitis, and meningoencephalitis. These conditions are typically associated with pregnancy, the new-born, the elderly, and those that are otherwise immunocompromised (1, 2). Although disease incidence is uncommon, mortality is as high as 30% (1). Given the severity of the disease, epidemiological surveillance and control of *L. monocytogenes* is important to ensure early detection of linked cases allowing timely intervention to protect public health and ensure the safety of the food chain. Whole-genome sequencing of *L. monocytogenes* is emerging as the primary means of molecular typing of isolates and allows epidemiological surveillance of strains from food sources and from clinical disease, thus facilitating detection of previously undetected links. This underpins the investigation of mechanisms that may influence disease pathogenesis (1, 3). To aid in the molecular epidemiological surveillance of the pathogen, the draft genome sequences of 25 *L. monocytogenes* isolates have been determined. The isolates were obtained from clinical cases of disease in Ireland between 2013 and 2015 and were submitted to the National Salmonella, Shigella and Listeria (human health) Reference Laboratory service at Galway University Hospital.

Whole-genomic DNA was extracted using the GenElute bacterial genomic DNA kit (Sigma Aldrich) per the manufacturer’s instructions. Library preparation and 250-bp paired-end sequencing was performed using the Illumina HiSeq 2500 platform (Microbes NG, University of Birmingham, UK). Raw reads were mapped to a reference genome using BWA-mem and *de novo* assembly was performed using SPAdes genome assembler. Contigs were reordered using Mauve aligner (v2.4.0). Prediction of putative open reading frames (ORFs) was performed using PRODIGAL prediction software (http://prodigal.ornl.gov/) and supported by BLASTx (4) alignments. Results of Prodigal/BLASTx were combined manually and a preliminary identification of ORFs was performed on the basis of BLASTp (4) analysis against a nonredundant protein database provided by the National Centre for Biotechnology (http://www.ncbi.nlm.nih.gov/). Using the ORF finding outputs and associated BLASTp results, Artemis (5) was employed for visualization and manual editing in order to verify and, where necessary, redefine the start of every predicted coding region, or to remove or add coding regions. The assignment of protein function to predicted coding regions was performed manually. In addition, the individual members of the revised gene/protein data set were searched against the protein family (Pfam) (6) and Clusters of Orthologous Groups (COG) (7) databases. rRNA and tRNA genes were detected using RNAMMER (http://www.cbs.dtu.dk/services/RNAmmer/) and tRNA-scanSE (http://lowelab.ucsc.edu/tRNAscan-SE/), respectively. COG category assignment (7) was performed by means of BLASTp (4) analysis against the COG database (8) for deduced proteins of all identified ORFs contained by the genomes of both *L. monocytogenes* strains that were sequenced as part of the current study, and of all publicly available *L. monocytogenes* strains.

### Accession number(s).

This whole-genome shotgun project has been deposited at DDBJ/ENA/GenBank. Accession numbers and basic genome information are presented in Table 1.

**TABLE 1:** Metadata for clinical *L. monocytogenes* Isolates described in this study

Isolate ID	NCBI BioSample no.	GenBank accession no.	Genome size (bp)	No. of contigs	Fold coverage
MQ130026	SAMN06309513	MUZG00000000	2,918,229	7	148.251
MQ130029	SAMN06309514	MVED00000000	3,024,609	7	160.075
MQ130032	SAMN06309515	MVEE00000000	2,920,693	7	202.454
MQ130033	SAMN06309516	MVEF00000000	2,976,451	13	132.789
MQ130037	SAMN06309517	MVFA00000000	2,948,715	9	200.801
MQ130042	SAMN06309518	MVEG00000000	3,039,834	7	120.659
MQ130058	SAMN06309519	MVEH00000000	2,977,371	8	143.952
MQ140011	SAMN06309520	MVEI00000000	3,133,360	12	71.1479
MQ140012	SAMN06309521	MVEJ00000000	3,115,895	11	123.737
MQ140025	SAMN06309522	MVEK00000000	2,938,002	7	112.804
MQ140029	SAMN06309523	MVEL00000000	2,923,309	8	130.18
MQ140030	SAMN06309524	MVEM00000000	2,881,390	10	115.135
MQ140031	SAMN06309525	MVEN00000000	2,919,535	8	142.567
MQ140032	SAMN06309526	MVEO00000000	2,898,261	7	101.054
MQ140033	SAMN06309527	MVEP00000000	2,918,397	8	125.069
MQ140034	SAMN06309528	MVEQ00000000	3,065,213	10	50.9738
MQ140035	SAMN06309529	MVER00000000	3,058,644	11	155.257
MQ150001	SAMN06309530	MVES00000000	2,923,292	8	136.199
MQ150004	SAMN06309531	MVET00000000	2,984,522	12	174.13
MQ150005	SAMN06309532	MVEU00000000	2,939,019	10	136.726
MQ150007	SAMN06309533	MVEV00000000	2,984,522	8	101.571
MQ150008	SAMN06309534	MVEW00000000	2,974072	8	126.961
MQ150011	SAMN06309535	MVEX00000000	3,025,843	8	75.1359
MQ150012	SAMN06309536	MVEY00000000	2,926,961	11	139.049
MQ150013	SAMN06309537	MVEZ00000000	3,009,735	13	147.238
